# Is aboriginal food less allergenic? A study on the IgE-binding capacity of egg white and yolk from modern and ancient chicken breeds investigated in a cohort of hen’s egg allergic children

**DOI:** 10.1186/2045-7022-1-S1-O19

**Published:** 2011-08-12

**Authors:** Gabriele Gadermaier, Matthias Egger, Claudia Alessandri, Michael Wallner, Peter Briza, Danila Zennaro, Adriano Mari, Fatima Ferreira

**Affiliations:** 1 University of Salzburg, Salzburg, Austria; 2 IDI-IRCCS, Center for Molecular Allergology, Rome, Italy

## Background

Hen's egg allergy ranks among the most frequent primary food allergies in children. We aimed to investigate sensitization profiles of egg allergic patients and compare in vitro IgE reactivities of eggs from two ancient chicken breeds with those from conventional laying hen hybrids.

## Methods

Egg allergic children (n=25) were subjected to skin prick test, double blind placebo controlled food challenge, and sensitization profiles to Gal d 1-5 were determined by allergen microarray. IgE binding and biological activity of eggs from ancient chicken breeds, i.e. Araucana and Maran and modern laying hen hybrids were investigated by immunoblot, ELISA and mediator release assays.

## Results

In our cohort, 48% of patients were sensitized to egg white and yolk, while 52% were reacting to egg white exclusively. In allergen microarray, Gal d 1 and 2 were identified as major allergens for all patients, whereas Gal d 3-5 displayed high sensitization prevalence only in patients reacting to both egg components. Mean egg white-specific IgE was significantly higher in patients displaying additional sensitization to yolk compared to yolk-negative individuals (6.31 μg/ml and 1.53 μg/ml, respectively). Eggs from ancient chicken breeds demonstrated reduced egg white/yolk ratios compared to those from conventional laying hen hybrids but did not differ in their allergen composition as determined by mass spectrometry. Accordingly, we observed no significant differences in IgE-binding and basophil mediator release assays comparing egg white and yolk from different chicken breeds.

## Conclusions

The onset of egg allergy seems to be mediated by egg white allergens expanding to yolk sensitization in later stages of the disease. Notably, our results on allergenicity and biological activity do not confirm the common assumption that aboriginal food might be less allergenic.

